# Use of multibeam imaging sonar for observation of marine mammals and fish on a marine renewable energy site

**DOI:** 10.1371/journal.pone.0275978

**Published:** 2022-12-14

**Authors:** Francisco Francisco, Anke Bender, Jan Sundberg

**Affiliations:** Division of Electricity, Department of Electrical Engineering, Uppsala University, Uppsala, Sweden; Havforskningsinstituttet, NORWAY

## Abstract

Environmental data is crucial for planning, permitting, execution and post construction monitoring of marine renewable energy projects. In harsh conditions in which marine renewable energy is harvested, integrated monitoring platforms comprising multibeam imaging sonar systems coupled with other sensors can provide multiparametric data of the marine environment surrounding marine renewable energy installations. The aim of this study was to test the possibilities of observing the occurrence of fish and marine mammals using a multibeam imaging sonar system deployed at a wave power test site. The results obtained from a ten-day data set proved the platform as suitable for long time underwater monitoring and also revealed that the occurrence of fish and marine mammals was distributed across characteristic time and space domains. Large fish [>0.4 m] frequently occurred at night-time and near the benthic zone. Small fish [<0.2 m] frequently occurred during daylight and within the pelagic zone. The occurrence of seals was periodically distributed along a daily cycle, with intervals of 1–2 hours between maxima and minima. In conclusion, the use of multibeam imaging sonar can be a reliable technique for the qualitative and quantitative observations of fish and marine mammals in general and at marine renewable energy sites specifically, including protected and economically important species.

## 1. Introduction

Today, both the renewable and non-renewable energy sectors are increasingly exploring resources within the marine environment, where the physical conditions are harsh. Over time, conventional monitoring technologies such as acoustic doppler profilers (ADCP), video cameras, remote operated vehicles (ROV), hydrophones, direct human observations, among others, have been used to survey the subsea environment e.g. [1, 2]. Notwithstanding, these methods have its limitation, especially when the survey have to be conducted in deep and murky waters, high seas and strong winds which are common conditions in which marine renewable energy is harvested. In such demanding conditions, safe, robust and reliable environmental data acquisition platforms are of great necessity for planning, execution and monitoring of the environmental impacts of marine renewable energy projects. Active underwater acoustic systems, such as multibeam imaging sonar (MBS), can be used as an essential tool for collecting data that would otherwise be acquired using e.g. optical cameras and visual observations. Sound Navigation And Ranging (Sonar) systems, in combination with other conventional monitoring methods, can provide the best and safest multi-dimensional data of the marine environment surrounding marine renewable energy devices and other submerged structures, especially in turbid and deep waters where diving and other conventional methods are risky and thus also expensive and impractical [3, 4].

The use of sonar systems with high operating frequency and high resolution (imaging sonar) enables engineers and scientists to gather detailed information of the underwater environment in a similar perspective as provided by optical and electromagnetic monitoring devices such as cameras and Radio Detection And Ranging (Radars) [5, 6]. Sonar, as an echo-ranging technology comprises a transducer, a multichannel receiver, and a display [7, 8]. Multichannel receivers are used to control the excitation of the transducer, reception of echoes, amplifications and signal processing. The display unit delivers an echogram for an echo sounder, or an acoustic image for a high-frequency imaging sonar [7–11]. Acoustic images consist of several echo records resulting from multiple beams that are spatially distinct, and the echo magnitude on each beam is generally encoded by intensity or colour [5]. MBS is the only measurement instrument used in this study to acquire acoustic images. On an MBS, the acoustic energy is emitted and received in multiple angles across-track swath (beam path), typically in a fan shape [12, 13]. Transducing elements are arranged in a 2-dimensional array. Generally, each element transmits pulses (signals) individually in a crescent order, and the echo is received simultaneously by all receivers [12, 13]. However, each echo is processed separately enabling a number of echo-beams to be formed by combining the outputs of the several arrays of transducing components with different phasing functions. This setup effectively steers the beam in several directions simultaneously. Furthermore, these components are arranged in a spiral configuration so that the beam pattern fills the field of view (FOV). A multibeam swath can contain up to 1500 beams arranged in angular sectors covering up to 180° of FOV. Modern MBS systems can operate in large frequency diapason and reach range resolution up to 1 cm and angular resolution of about 0.2° [4, 14, 15]. The use of several narrow beams with a minimized transmit pulse (beam spacing) maximizes the effective sampling volume covered in the entire swath in a single ping. Multibeam imaging sonar systems also have limitations operating on short range only within 100 m, high sensitivity to noise and require demanding signal processing [9, 10]. Very high operating frequencies limit the range to less than 100 m in most of MBS systems. Background noise generated by seabed echoes will also negatively interfere with the main signal, particularly when the target is located at longer distance than the bottom depth [16, 17]. Bubbles and turbulence within the swath can also cause intense noise. Signal processing on an MBS is complex and data analysis is still time consuming as large volumes of data are quickly generated.

In order to minimize risks associated with subsea work, it is important to monitor the underwater environment and the impact on installations, operation and maintenance of energy converting devices such as generators, subsea substations, underwater cables etc [16]. Subsea monitoring of the marine environment is also a common requirement from authorities for consent of permits, not the least to investigate eventual effects on protected and/or economically important species. The costs and risks of such complex and continuous monitoring tasks can be significantly lowered while ramping the quality of the data, by utilizing multiparametric monitoring platforms based on sonar systems. Such platforms are becoming the preferential monitoring tool across the marine renewable energy sector. For example here [18–22] have used sonar systems to monitor fish and mammal interaction with tidal turbines and wave energy converters; [13–22] used multi-beam sonar systems to map seabed within hydrokinetic sites. The increasing exploration of the ocean environment for a wide range of resources (e.g., oil, gas, fisheries, new molecules, and soon, minerals) raises global concerns about potential ecological impacts. Besides the use in the renewable energy sector, multiparametric monitoring platforms could be used to fulfill baseline assessments, monitoring strategies, and environmental impacts assessments to evaluate natural spatial and temporal variability and to develop mitigation and restoration strategies beyond traditional monitoring methods [23, 24]. A multifunctional environmental monitoring platform based on sonar systems have been developed. After series of tests, this platform can be utilized for a variety of subsea monitoring tasks including marine fauna monitoring, inspection of water column, seabed and energy devices. The main sensors on-board the monitoring platform were a MBS, a split beam (SBS) and a dual beam (DBS) sonar systems as well as underwater cameras (UWCs).

Historically, subsea monitoring techniques involved divers, electromagnetic based instrumentation, or low frequency acoustic systems, but also various methods of test fishing and the use of dredge and trawls. Divers as well as electromagnetic instrumentation have limited performance underwater due to safety and signal absorption, respectively [3, 25, 26]. Low frequency acoustic systems have limitations of angular resolution, therefore cannot replace divers and electromagnetic instruments such as video and laser cameras [26, 27]. On the other hand, multibeam imaging sonar can operate safely and function as a complementary tool to cabled observatory systems [28, 29] in dark and deep waters providing underwater acoustic images of targets. It can be hypostasised that imaging sonar systems can be used supplementary to monitor marine animals, and study the behaviour, occurrence, and biomass of individuals within the sonar FOV [24].

The objectives of this study were to test the performance of the platform by observing the occurrence and behaviour of fish and marine mammals within a wave power test site. Fish and Cetaceans are species groups that could be affected by marine energy installations. We did this by using a multibeam imaging sonar integrated into a standalone platform and to further improve the technical knowledge regarding the capabilities of the MBS for quantitative and qualitative monitoring of marine animals as well as refining the data acquisition and processing framework. The present study, similar to [30], is one of the first documented in which a MBS was used to monitor the subsea environment surrounding wave energy converters (WECs). This study also complements previous studies within the wave power research on the Swedish West coast, such as the use of hydrophones, video cameras (e.g. [7]), and quantitative sampling (e.g. [8]) to gather environmental data.

## 2. Materials and methods

### 2.1 Study site

**Fig 1:**
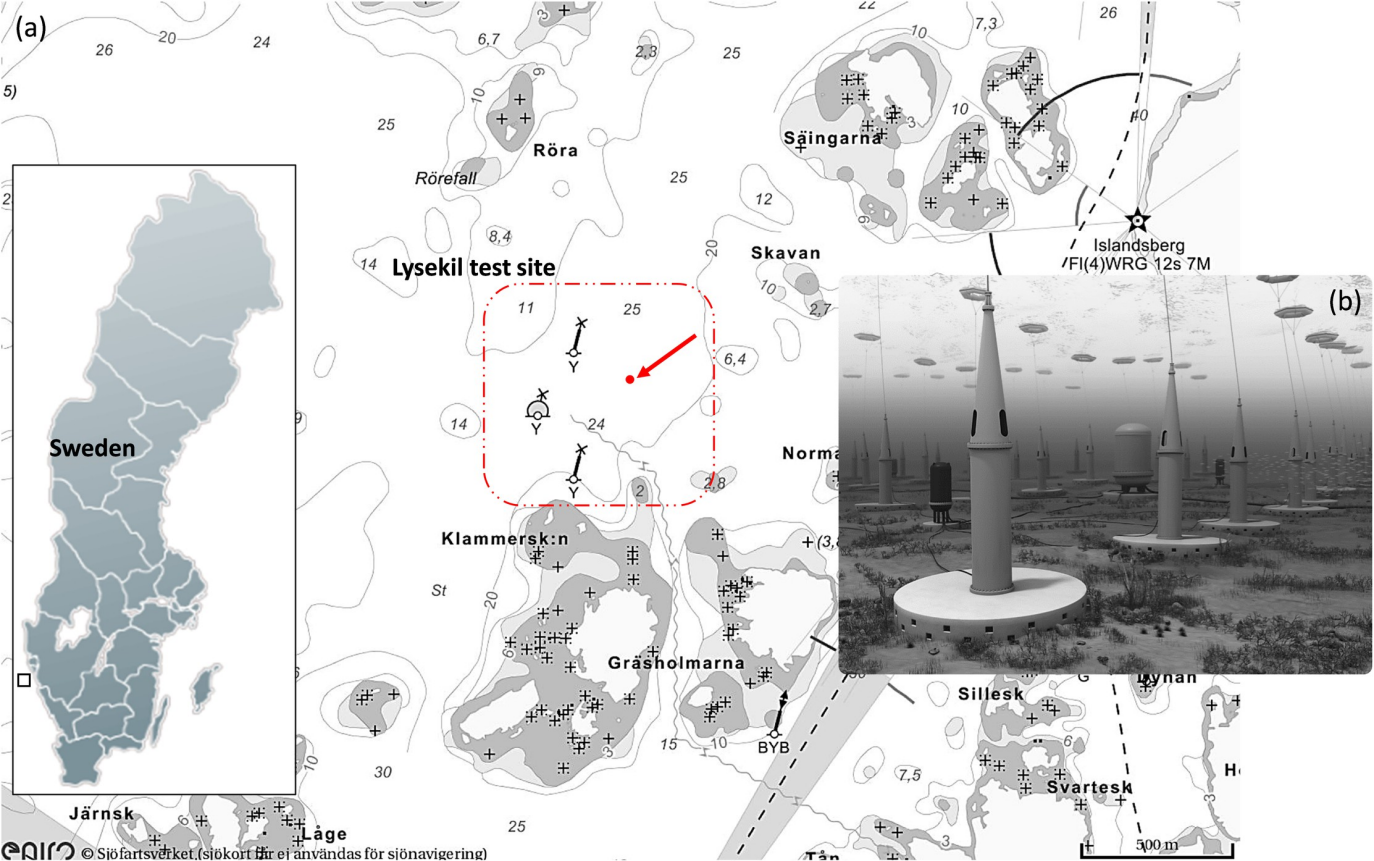
Location of the Lysekil research site on the west coast of Sweden. (a) The arrow indicates where the sonar platform was deployed. (b) Computer rendering of the subsea environment within the test site.

The survey was conducted at the Lysekil wave energy research site located on the west coast of Sweden (Fig 1A), within the Skagerrak strait. The site has an average depth of 25 m, the most frequent sea state has an average wave power value of 2.6 kW/m [0 kW/m to 25 kW/m], significant wave height of 0.7 m [0.1 m– 3 m] and an energy period of 5 s [2 s– 7 s] with predominant direction of propagation from the west [31, 32]. The salinity varies from 24 ppm in the surface to 33 ppm near the benthic. The mean sea surface temperatures vary seasonally from 0°C– 20°C and is approximately 4°C annual average near the benthic. Since the commissioning of the test site in 2004 several Uppsala University WECs have been deployed in conjunction with other support equipment [33]. The UU-WEC technology (Fig 1B) is a point absorber consisting of a submerged linear generator connected to a heaving buoy [34]. An onsite submarine substation connects the WECs to an onshore grid connection point [35]. Several monitoring studies were conducted before to assess the impact of the UU-WEC to the marine environment. Artificial reef effect, underwater radiated noise are few of the effects so far studied during operational phase [26, 27].

### 2.2. Ecological characteristics within the experimental site

MBS can distinguish a species only based on peculiar traits of its morphology (morphospecies), being taxonomic resolution usually at higher level; e.g., Order and frequently occurring marine species on the Swedish west coast [36] are listed Table 1. Common pelagic fish species in the area are e.g., the Atlantic cod, Atlantic herring, European sprat, and Atlantic mackerel [36–38]. Harbour and grey seals are common marine mammals observed in this area whereas dolphins and orcas (Cetaceans) are more rare visitors [36–38]. Harbour seals take a role as top predators of mainly flatfish, cod and herring amongst others and by that seals are contributing to a top-down control of the food web of the Skagerrak [39, 40]. Seals usually increase swimming speed with several strokes of the hind flippers just prior to attempting a capture [38, 41]. This behavior is hereafter referred to as hunting.

**Table 1:** Common species of interest on the Swedish west coast [35].

Species	Size	Swimming & Behaviour	
		Mode	Activity
Atlantic cod	60 cm– 1.2 m	Shoaling	Near the benthic at nigh time
*Gadus morhua*			
Atlantic herring	24–45 cm	Schooling	At high energy environments
*Clupea harengus*			
European sprat	8–16 cm	Schooling	At pelagic inshore
*Sprattus sprattus*			Diel migration, deep water–day; mid-surface waters-night.
Northeast Atlantic mackerel	30–66 cm	Schooling	Fast swimming pelagic. Closer to shore in summer and spring.
*Scomber scombrus*			
Grey seal	Males 2–3.3 m avg. 2m	Diving and swimming	V-shape diving to near the seabed; slow swimming near the bottom. Foraging dives are square/U-shaped.
*Halichoerus grypus*	Females 1.6–2 m avg. 1.8 m		
Harbour seal	Males avg. 1 m	Diving and swimming	Square/U-shaped—faster descending, ascending and longer time in the bottom;
*Phoca vitulina*	Female avg. 1.7 m		
			Two-phased dives–when the dive period is done two or more distinct phases at different depths. Commonly when following schools of fish and also when “scanning” for prey.
Orca	Males 6–8 m	Diving and swimming	Square/U-shaped, faster descending/ascending and longer time submerged in the few meters below the sea surface.
*Orcinus orca*	Females 5–7 m		
	Dorsal fin 0.9–1.8 m		

### 2.3. Data collection

**Fig 2:**
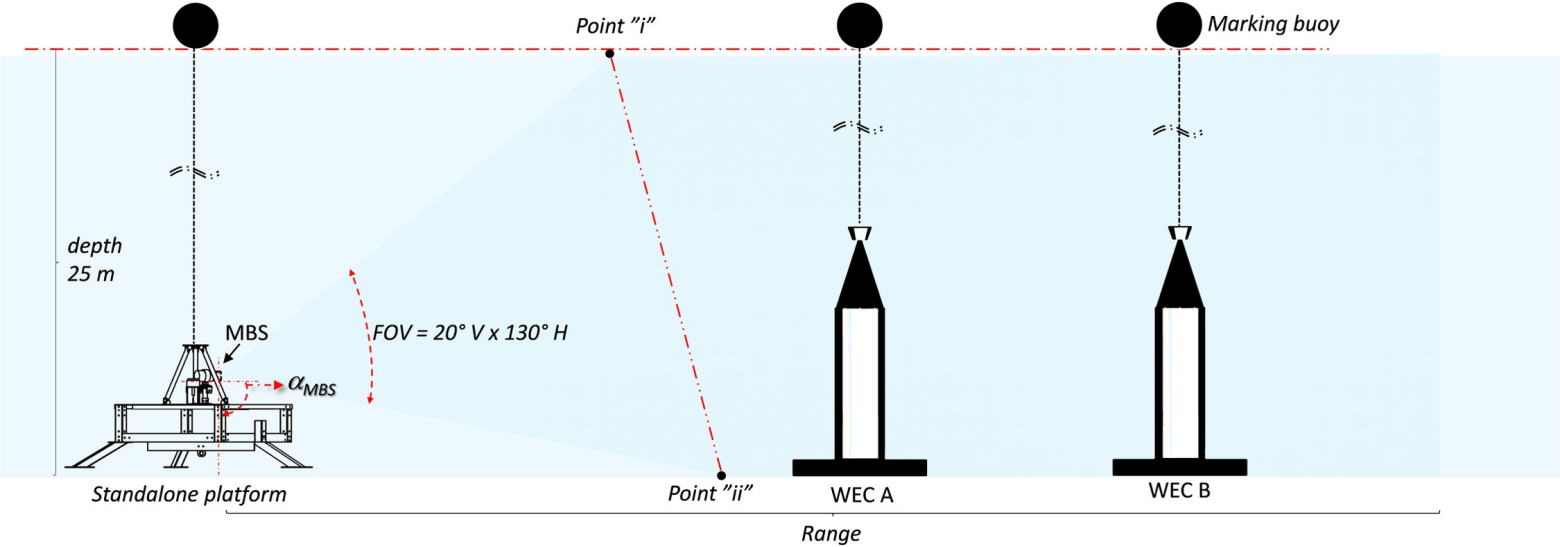
The standalone monitoring platform: (a) The multibeam imaging sonar (MBS) together with split beam sonar (SBS), the three boxes contain batteries, controllers and an on-board computer; (b) The standalone platform deployed at the seabed.

**Fig 3:**
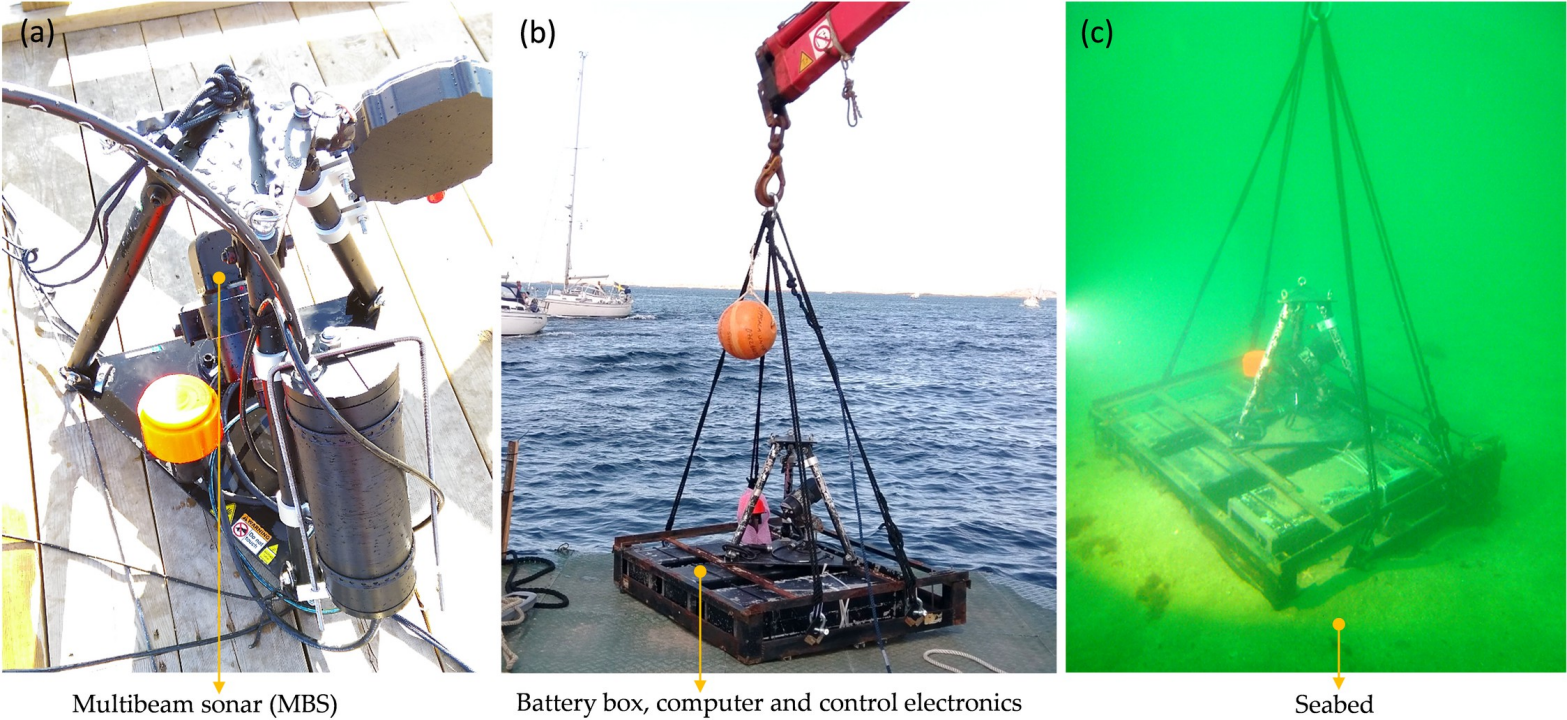
Lateral view-scheme to facilitate the interpretation of the acquired multibeam data and the resultant acoustic images. The multibeam sonar (MBS) was located at 24.3 m of depth at a distance of 30 m and 50 m from WECs A and B respectively. The field of view (FOV) opens in 20° vertically and 130° horizontally. The MBS pitch angle (αMBS) was set to 10°.

The data was collected using the standalone platform (Fig 2), comprising a submerged tripod (1.7 m H x 1.8 m W, 250 kg) that houses the sensors, computer, control electronics and a battery bank (Table 2). The platform was deployed from a boat onto the seabed at 25 m depth pointing towards two WECs. A small buoy marked the deployment location and was connected to the submerged unity through a wire (Fig 3C).

**Table 2:** Technical specifications of sensors and components used in this study.

Component	Specification	Component	Specification
Computer	Clock: 1.0 GHz	Multibeam imaging sonar (MBS)	Frequency: 0.9 MHz (operational)
Artigo	RAM: 4GB DDR3		Number of Beams: 768
	HDD: 2TB (storage memory)	Blue view	fps: up to 50Hz (sample frequency)
	VGA: FULL HD 3D		FOV: 132x20 (field of view)
	Input Voltage: 12 VDC		Resolution: 0.18° / 2.54 cm
			Maximum range: 100 m
Battery bank	12 V, 7.8 kWh		

### 2.4 The survey setup

**Fig 4:**
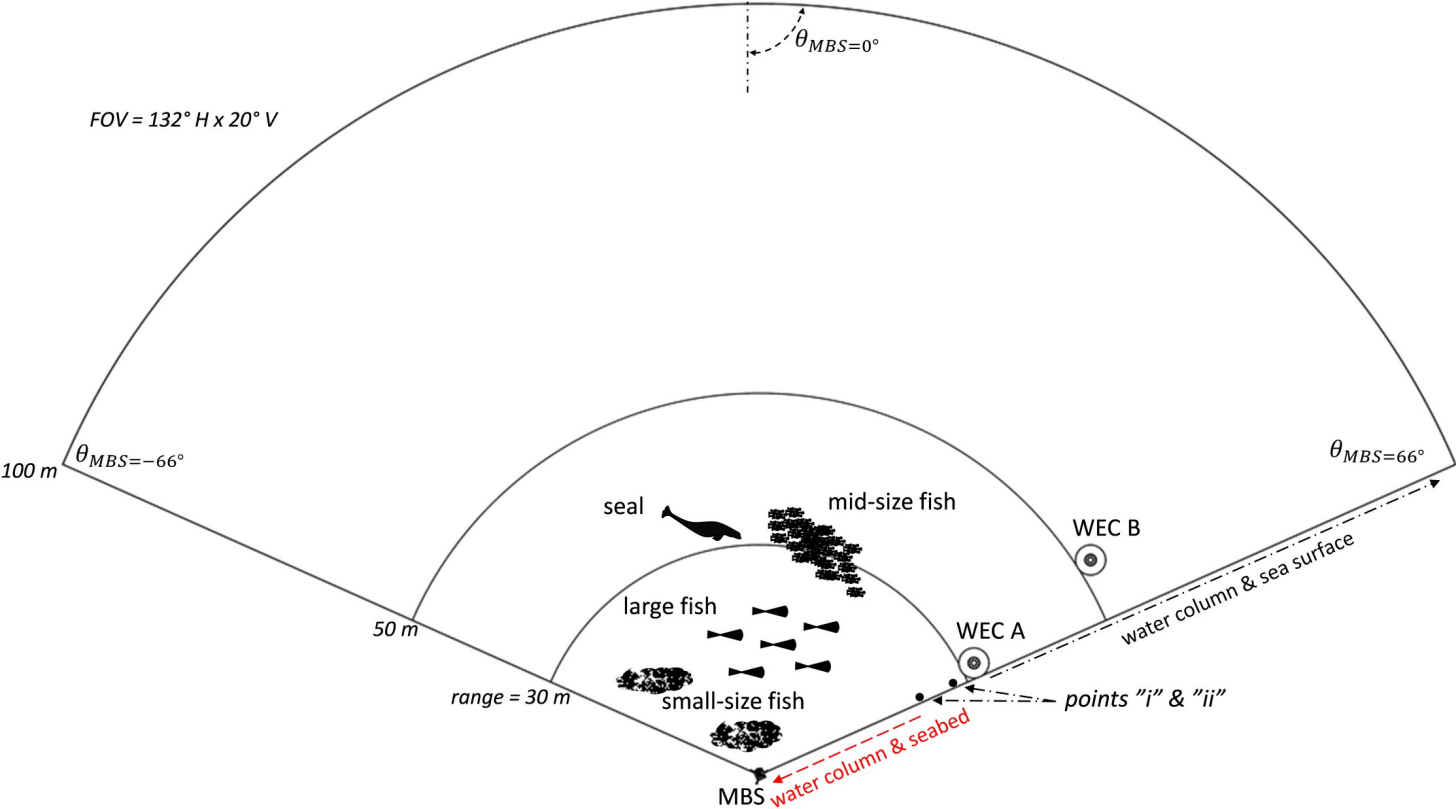
Top view of the sonar field of view (FOV). This scheme facilitates the interpretation of the acquired multibeam (MBS) data and acoustic images. The maximum width of the FOV was 82 m or 130° (*θ*_*MBS*_ = -66° to 66°) and the maximum range was 100 m. Most of the classified targets were detected within 50 m of range. The MBS was located at a distance of 30 m and 50 m from WECs A and B, respectively.

The MBS data was acquired between 23rd August and 1st September 2016. The MBS was deployed with the sonar transducer orientated upwards at height of 0.7 m above the seabed, with pitch angle of αMBS = 10° (Fig 3). The FOV of the MBS covered two UU-WECs, WEC A at 30 m of range and yaw angle of *θ*_*MBS*_ = 58°, WEC B at 50 m of range with *θ*_*MBS*_ = 52°, and part of the seabed and sea surface. The FOV intercepted the sea surface plane at 25 m of range which is also equivalent to 25 m (point “i” Figs 3 and 4) of local bottom depth. It also intercepted the seabed plane at point “ii”. From point “i” onwards the data mostly contained backscatter insonification (irradiated acoustic energy) from the water column and sea surface. From point “ii” backwards the data mostly contained backscatter data from the water column and seabed.

**Fig 5:**
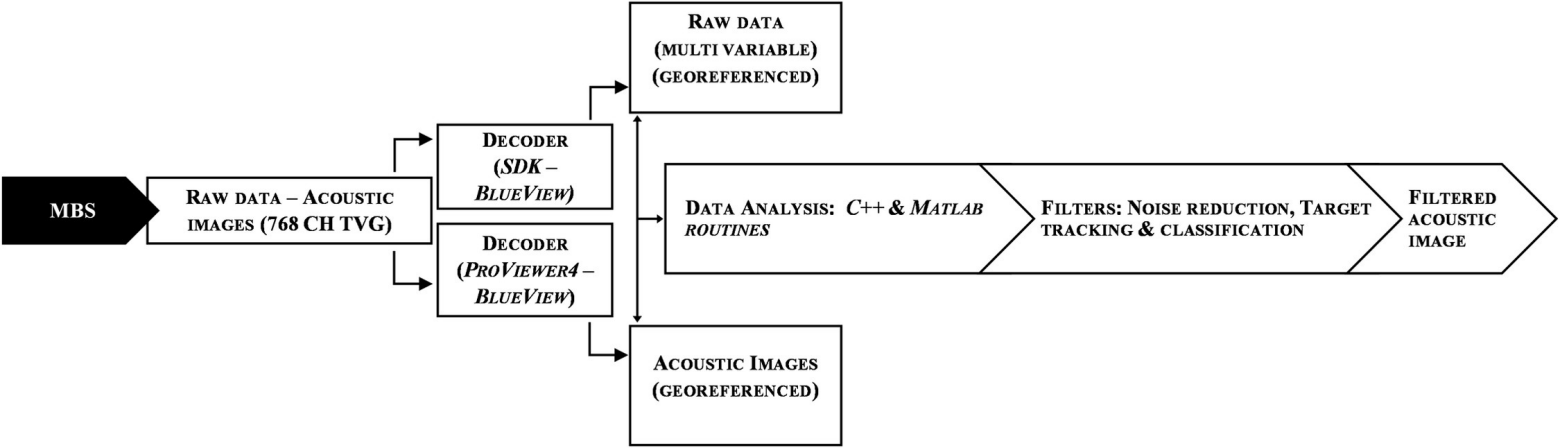
Architecture of the data acquisition and processing for detecting, tracking and classifying underwater targets of interest. The MBS signal is promptly processed by the SDK/ProViwer software running on C++ programming environment. The acoustic images are then analysed and filtered using the SDK/ProViewer and Matlab—Image processing Toolbox [42].

The sonar was programmed to operate autonomously. Each observation comprised sequences of 1199 samples at 1.25 Hz lasting a total of 16 min. The time lag between observations were set to 47 min. This sampling plan was the optimized around volume of data versus memory and battery capacity versus survey duration. In total 260 observations (311740 samples) of targets were conducted within the period of ten days. The MBS uses the BlueView SDK 3.6 and ProViwer4 software for data acquisition and processing. Each sample equals to a ping, which essentially is an echo comprising an aggregate of 768 beams that form an acoustic image. Each ping contains information of backscatter intensity of insonified targets, range, georeferenced, and velocity data. However, this study only used backscatter intensity and range data for analysis, through a routine described on Fig 5. Acoustic images were submitted to further analyse using supervised classification of the backscattering intensity values using Matlab. A valid set of targets or valid pixels were selected as the representation of a specific class of targets. The image processing algorithms used this information (training) as a reference to be used to classify targets on all other images. This process was repeated and improved several times to make the target classification algorithm more reliable. The target classification capability did not got affected by the range, i.e., by the distance from the MBS.

The measure of target length (size) and range might have been affected by precision and reading errors, while the manual and automatic counting of target was affected by accuracy errors. The precision/reading error was estimated to be ±0.5 cm of measured length. Estimating the accuracy of the measurements would have required a true value or expected (reference) value. Since there were no reference values to compare with, the uncertainty was characterized by bootstrapping considering omission, coordination and double-counting errors.

## 3. Results

### 3.1 Occurrence of targets

**Fig 6:**
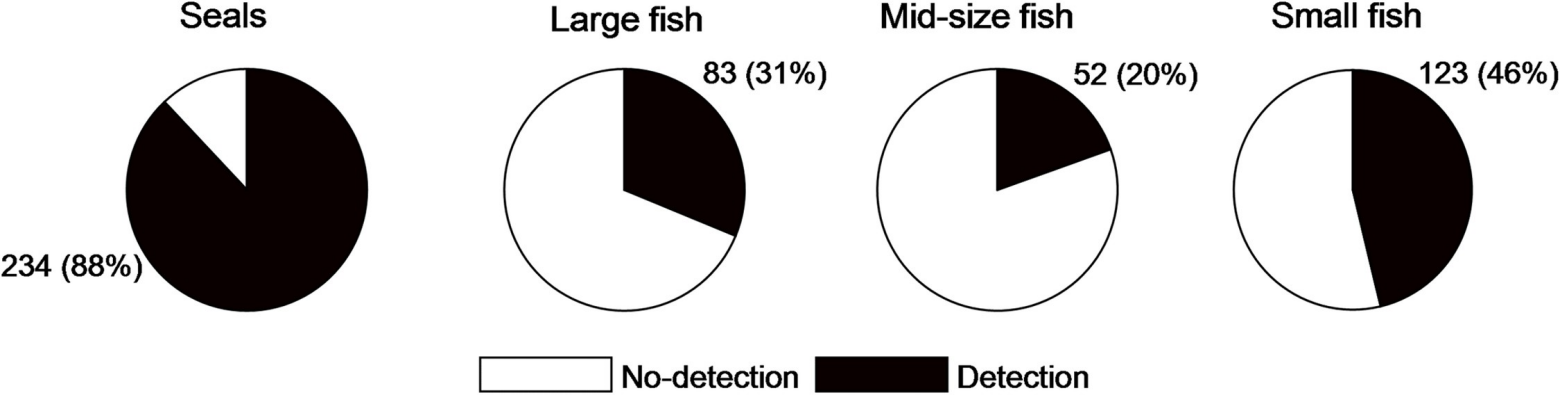
Total number of observations versus number of observations that resulted in target detection. For example, on a total of 260 observations 234 contained seals, and 32 did not contain seals.

A total of 260 observations of targets were analysed, based on a total of 4160 min of data containing 311740 acoustic images. 826 targets were identified as harbour or grey seals, 4 targets identified as a large marine mammal; 1607 targets identified as large fish; 58 and 76 targets were identified as schools of mid and small-size fish, respectively. The number of counted targets may not reflect to number of individuals; instead, it most likely reflects the counted number of detected target whether the target is the same or different individual or group. Within this data set, seals were the most detected target in 234 observations out of 260 observations. The second most detected target were schools of small-size fish: 214 out of 260, followed by large fish: 143 out of 260. The least occurring target was schools of mid-size fish: 52 out of 260 (Fig 6).

**Fig 7:**
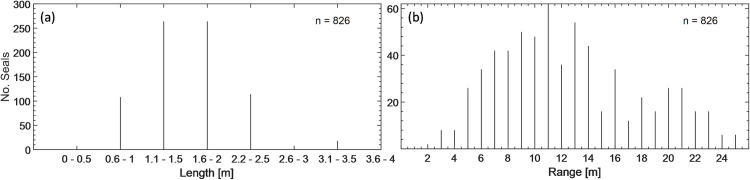
Distribution of occurrence of seals in relation to (a) length and (b) detection range. Seals measuring 1.1 m to 2 m were the most frequent and were mostly detected within 6 m to 16 m of range.

**Fig 8:**
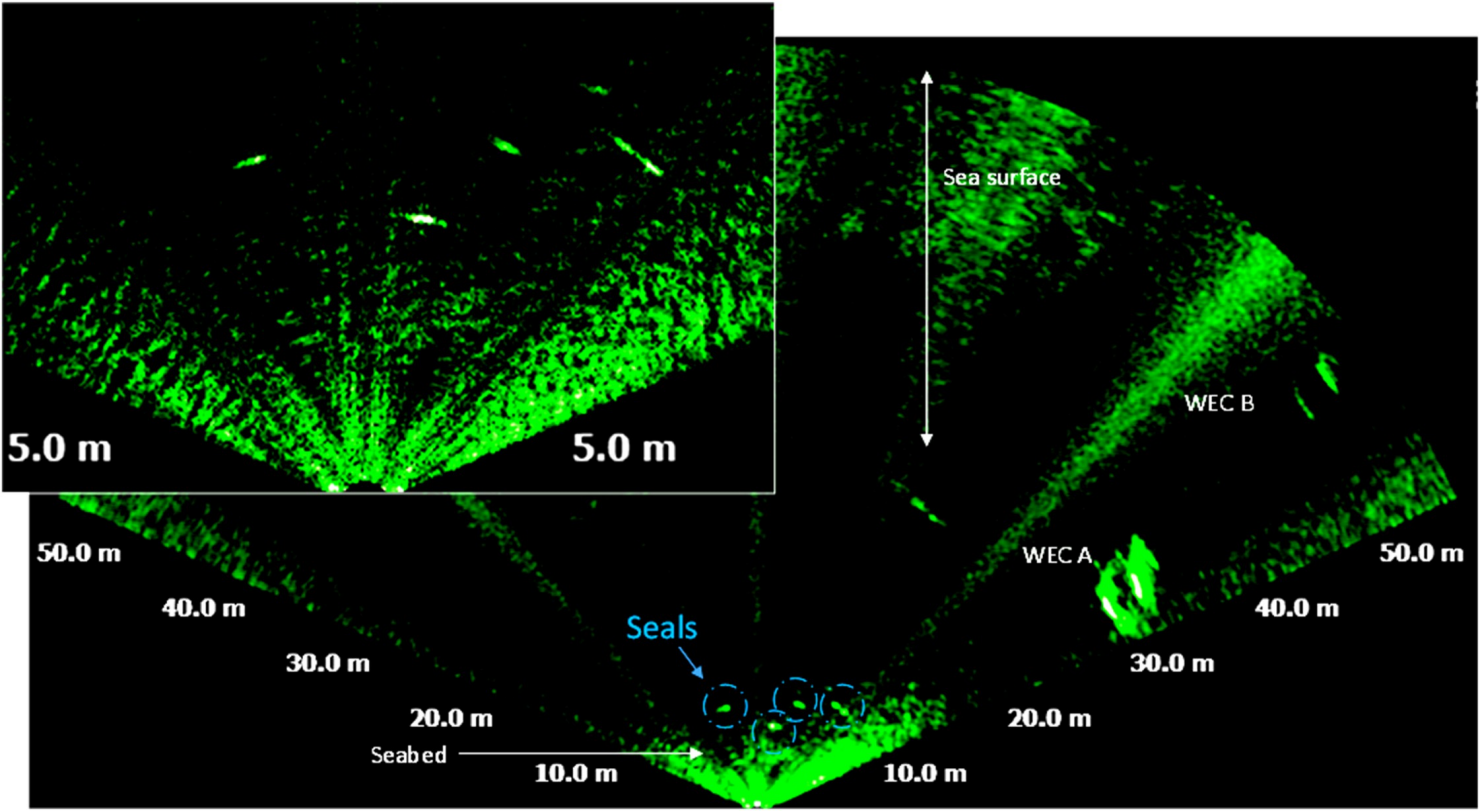
Acoustic image acquired on 26/08/16 at 09:20 UTC showing a group of four seals, measuring between 1.2 m to 2 m of length, detected at 6 m to 11 m of range from MBS and at distances between 20 m and 30 m from WEC A.

The most frequently occurring seals measured between 1 m– 2 m, followed by seals with lengths of ca. 2.1 m– 4 m (Fig 7A). Seals with length between 1 m– 2 m were frequently observed swimming in groups of up to six individuals but also occurred alone (Fig 8). Larger seals with lengths between 2.1 m to 4 m were frequently swimming solo or in groups of two individuals. Seals were mostly observed within 5 m to 20 m of range (Fig 7B).

**Fig 9:**
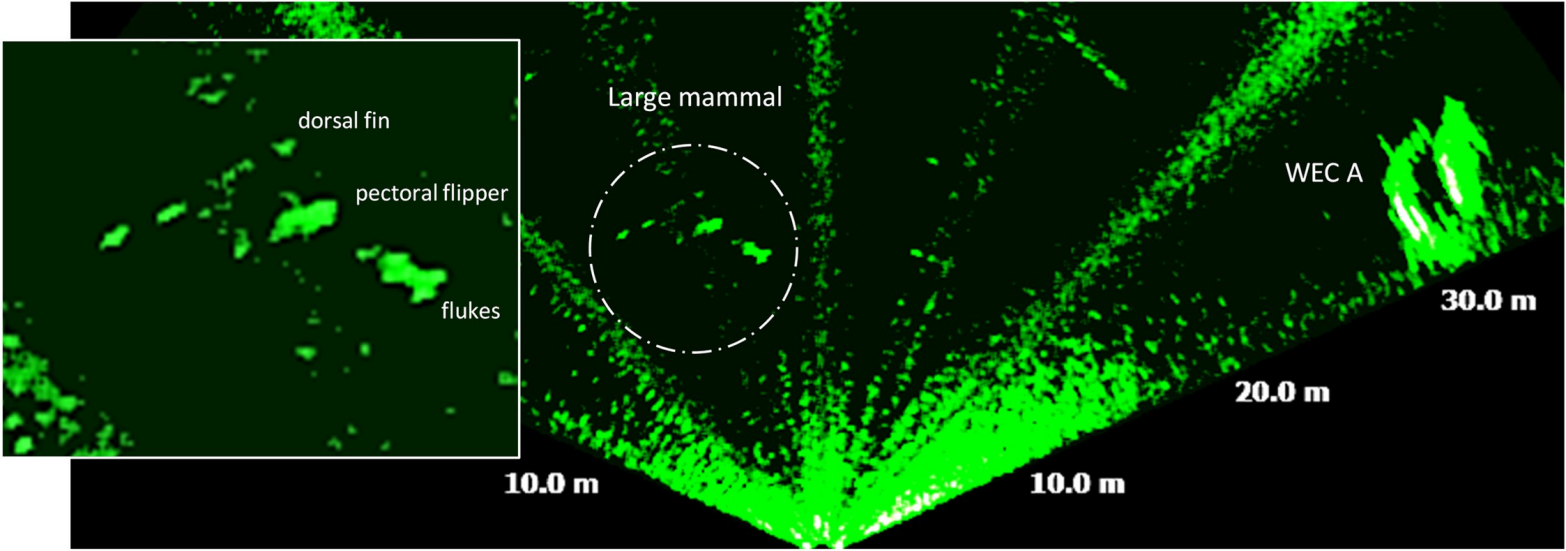
Acoustic image acquired on 28/08/16 at 19:39 UTC showing a large marine mammal (possible orca) located at 15 m of range, with 7 m of length, and a dorsal fin measuring 1.3 m of height.

On four occasions, the MBS detected large targets with length of between 4 m and 7 m. These targets displayed an ellipsoidal, streamlined shape and were observed at ranges of 5 m– 6 m, and at distances of at least 20 m from WEC A. The shape of these the targets suggests that they were large cetaceans. For example, Fig 9 show a target measuring 7 m in length and 3 m in height and where the dorsal fin alone measured approximately 1.3 m.

**Fig 10:**
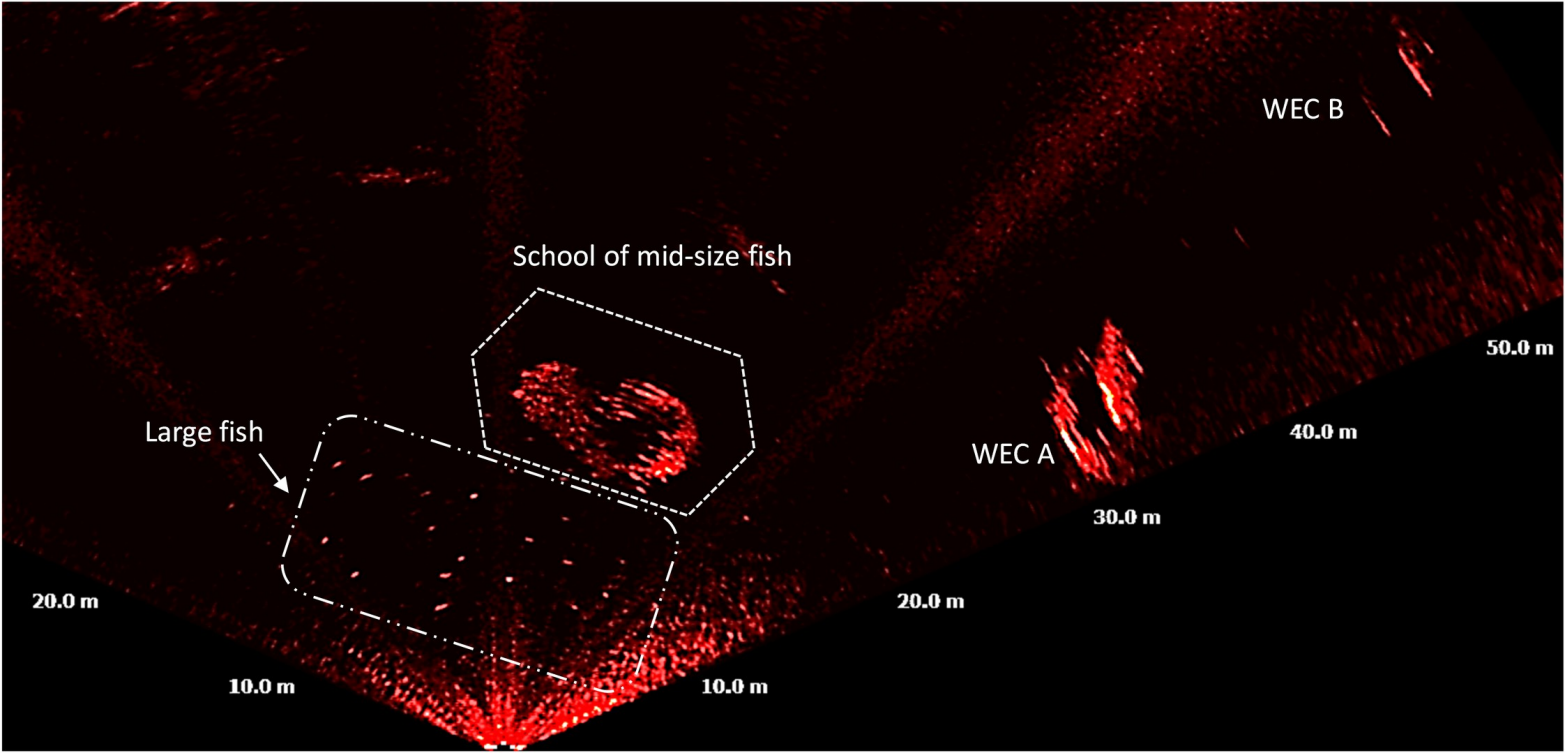
Acoustic image acquired on 26/08/16 at 19:26 UTC, showing a school mid-size fish and at least 20 large size fish. The school measured 12 m of diagonal length and the individual large fish measured between 0.4 m and 0.9 m.

Smaller sized targets were unambiguously fish and were categorised in three size categories, large fish [>40 cm– 90 cm], mid-size fish [20 cm– 40 cm] and small-size fish [< 20 cm]. These targets crossed the FOV at a variety of ranges from the MBS, from 0.6 m to over 30 m, and from the benthic zone to the surface (Fig 10). Large fish frequently occurred near the benthic and up to the pelagic zones, at ca. 15 m of depth. Mid and small-size fish were observed mostly in the pelagic and pelagic zones, in schools of 5 m to 14 m of length (diameter).

### 3.2 Diurnal variability

**Fig 11:**
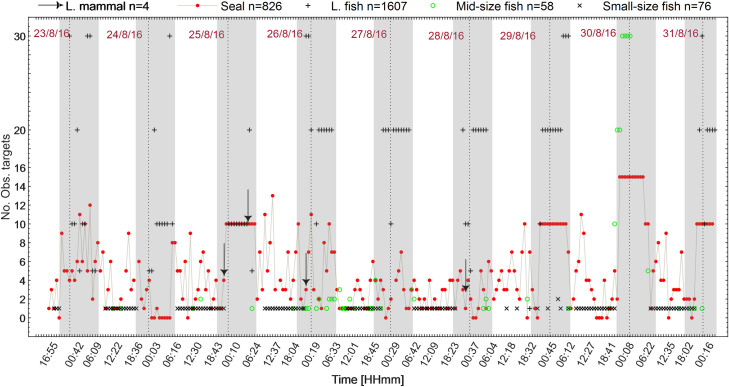
Time series of the observed targets starting from the time of deployment (23-08-2016) until the time of retrieval (01-09-2016). Small and mid-size fish were observed and counted in schools. The occurrence of small-size and large fish had diurnal and nocturnal patterns, respectively. Seals occurred with a random trend along the time. The grey shaded areas represent the night-time.

The occurrence of the observed targets was not uniform over time (Fig 11). Although not a primary aim of this study a clear result is that some of the target sizes occurred more frequently during daylight others during night-time. Large fish occurred entirely during night-time while schools of small-size fish were observed entirely during daylight (Figs 11 and 13).

**Fig 12:**
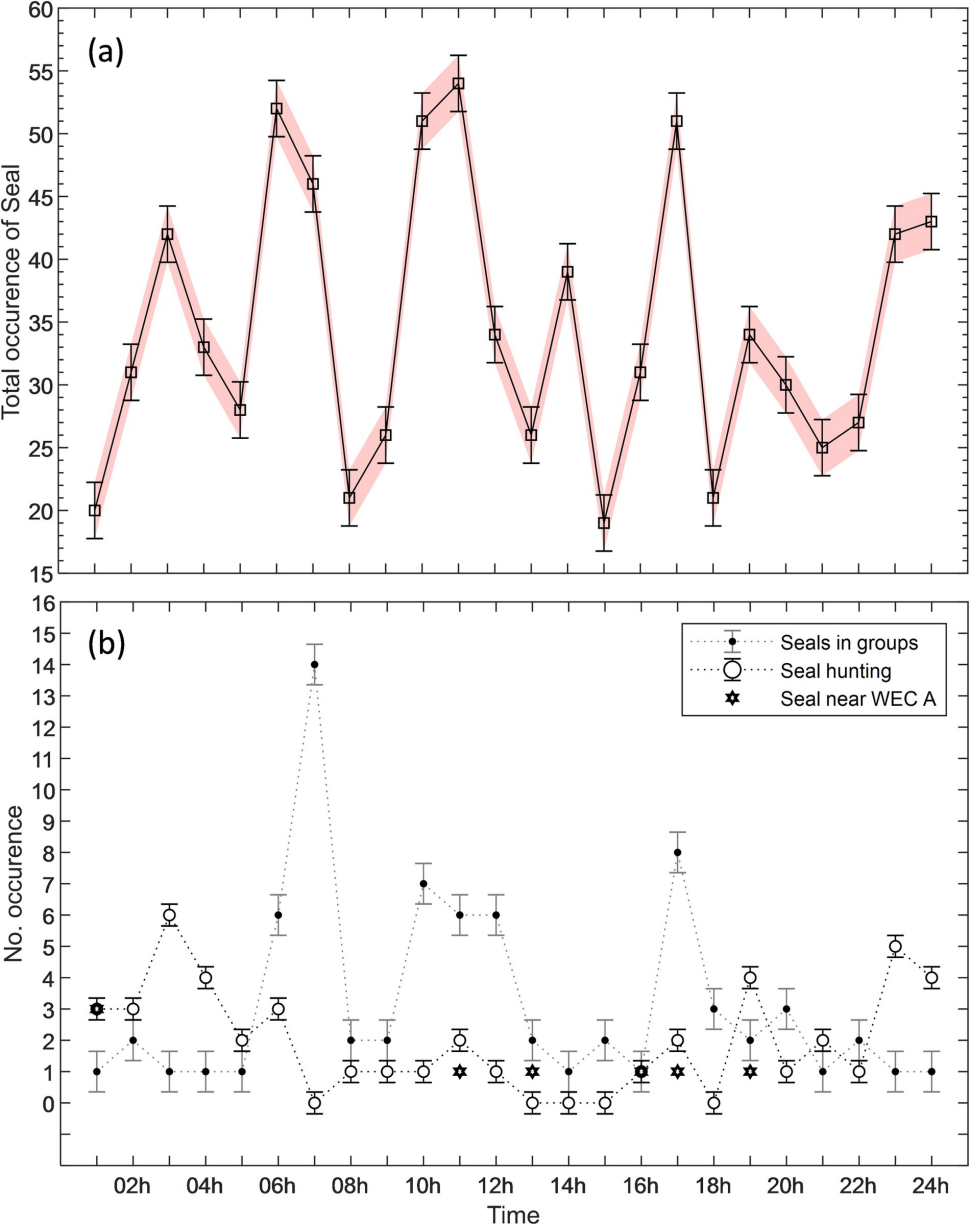
Temporal variability, occurrence, and behaviour of seals. (a) Total occurrence of observed seals per hour; (b) Total occurrence of seal swimming in groups, engaged in hunting, and swimming close to WEC A. The standard error is represented by the error bars with caps.

Seals occurred throughout the entire daily cycle, and with a periodicity of one to two hours (Figs 11 and 12A). Seals were observed moving both slowly and fast. The fast movements, observed on 47 occasions, were mostly in direction towards schools of fish. The hunting behaviour was reaching peaks at night-times (Fig 12B). Harbour seals were frequently observed swimming in groups of 2 up to 6 individuals. The regimentation behaviour occurred mainly during daytime (Fig 12B). Only eight observations of seals moving at distances shorter than 5 m from the WEC A were recorded (Fig 12B).

**Fig 13:**
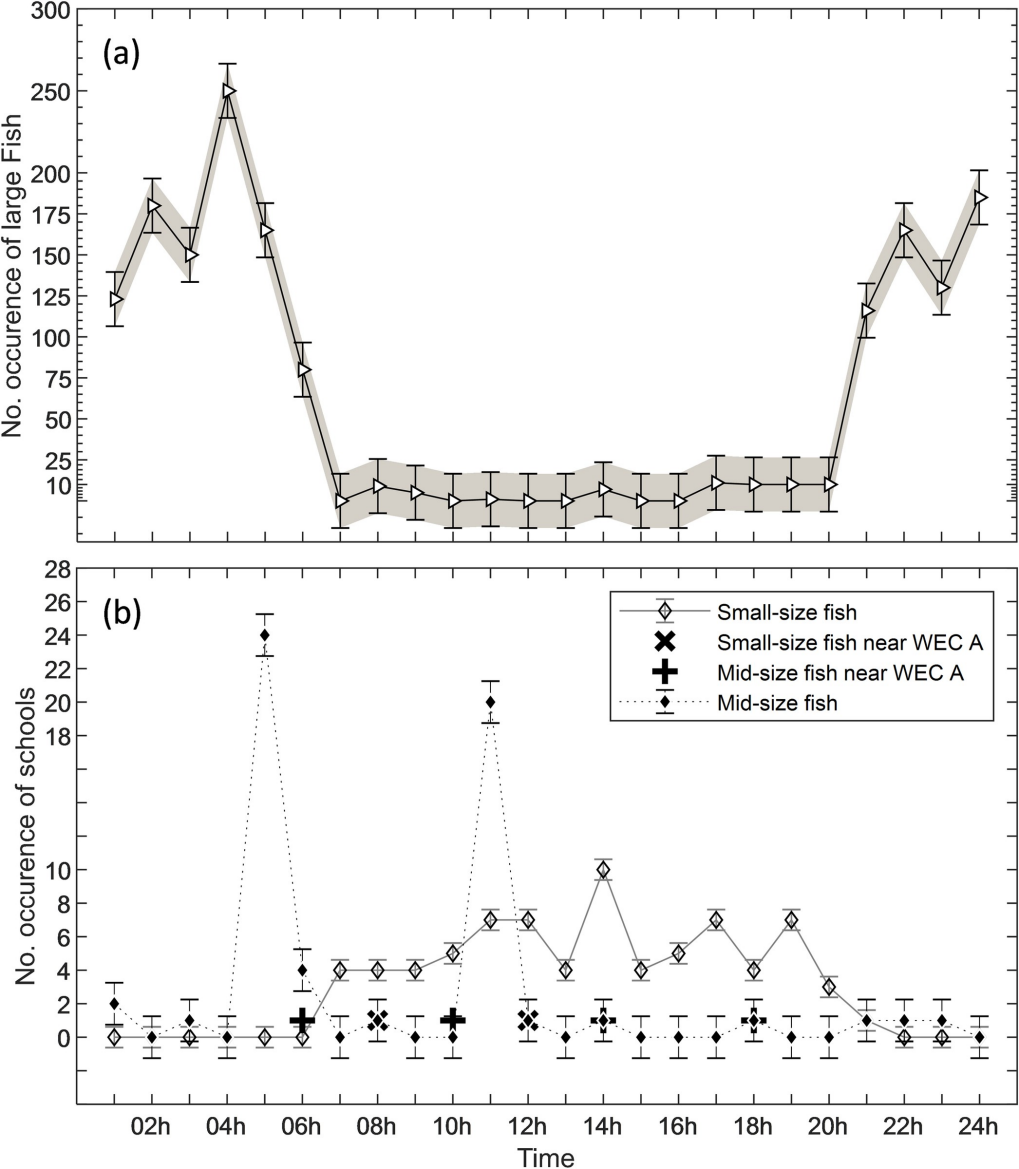
Temporal variability, occurrence and behaviour of fish. (a) Total occurrence of large fish (> 40 cm); (b) Total occurrence of schools of mid-size (20 cm to 40 cm) and small fish (< 20 cm). The standard error is represented by the bars with caps.

**Fig 14:**
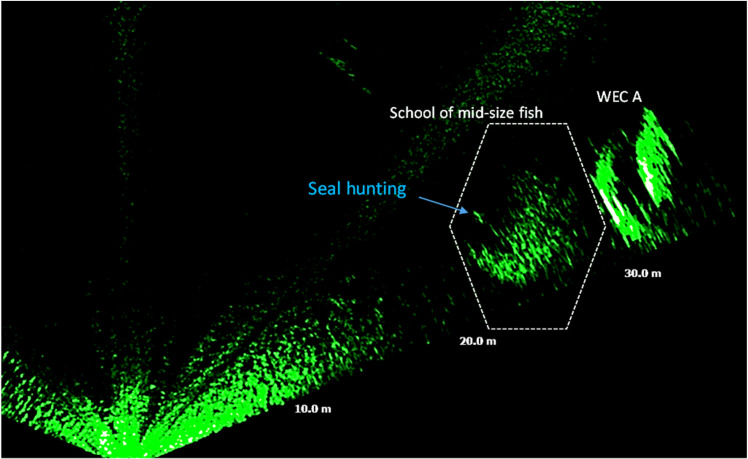
Acoustic image acquired on 27/08/16 at 09:22 UTC, showing a school of mid-size fish passing close to WEC A. A hunting seal measuring 2.5 m of length, was detected hunting and diving into the school.

**Fig 15:**
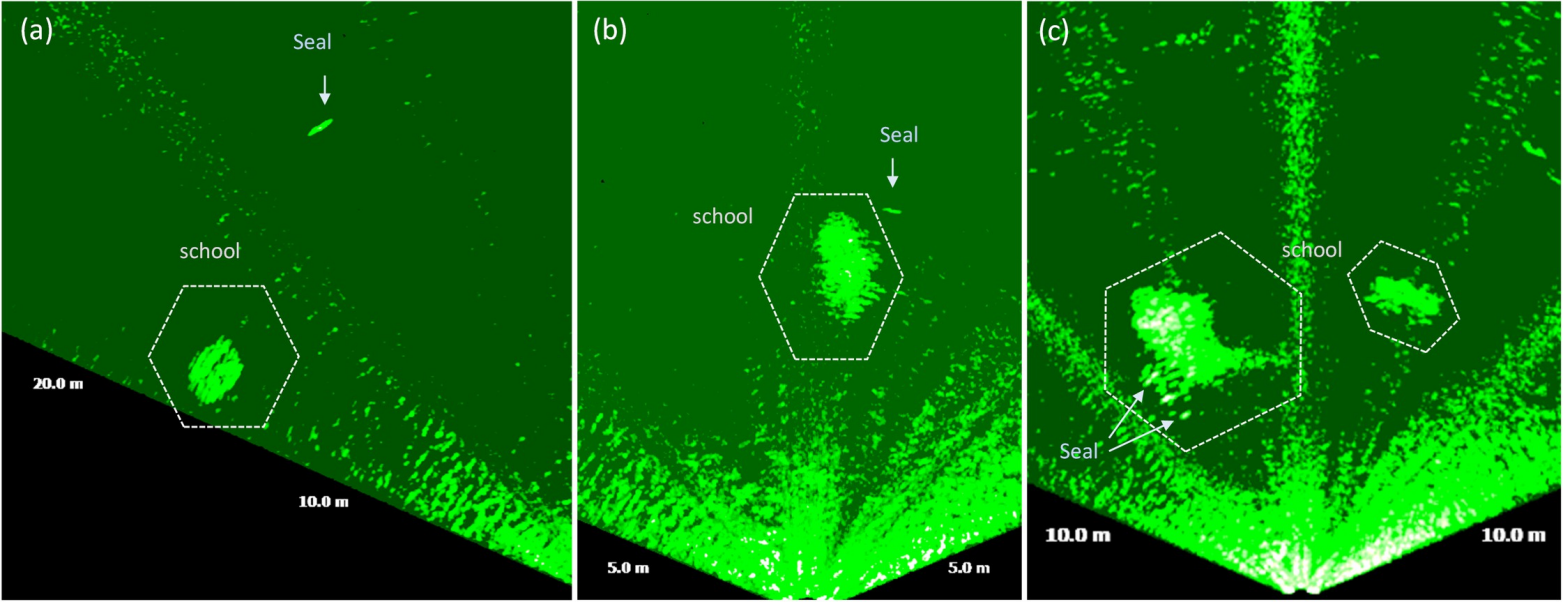
Acoustic images showing seals diving into schools of mid-size fish: (a) a school with 2.5 m of diameter and a seal of ca. 1.3 m of length, observed on 27/08/16 at 16:20 UTC; (b) school with ca. 3.5 m of dimeter and a seal of ca.1 m, observed on 27/08/16 at 09:18 UTC; (c) school with ca. 10 m of diameter and seals of ca. 1 m, observed on 27/08/2016 09:20 UTC.

Large fish occurred during night-time and at dawn with activity starting at ca. 20 UTC and lasting until approximately 6 UTC with a peak at 5 UTC (Fig 13A). Schools of mid-size fish occurred irregularly during the day (Fig 13B). Schools of small-size fish were observed exclusively during daylight, from 7 to 20 UTC. Schools of small fish were also detected on six occasions passing at distances of 2 m to 10 m from WEC A (Fig 14), during daylight (Fig 15), presumably when seals were actively hunting.

## 4. Discussion

This is one of the first studies carried out at an offshore wave power farm, in which the use of multibeam imaging sonars proved to be a capable and reliable technique for monitoring the marine environment surrounding WECs. Using a standalone platform deployed for ten days at the Lysekil research site, made it possible to do both, qualitative and quantitative data collection of occurrences, size and behaviour of fish and marine mammals, also revealing characteristics in distribution in time and space. Diel variation in occurrence of fish is well known, both in fresh water and oceanic conditions and has been studied in relation to tidal and instream renewable energy conversion [43–45]. In our study large fish frequently occurred at night-time and near the benthic zone, while small-size fish frequently occurred during daylight and within the pelagic zone and mid-size fish occurred in large and dynamic schools.

Seals are also known to show diurnal patterns and has also been studied in relation to tidal turbines (e.g. [38, 46, 47]). Our results show that 1 m– 2 m long seals occurred more frequently than the seals of 2 m– 4 m of length. The small sized seals also occurred more frequently in groups with more than two individuals. The small size seals were doubtless harbour seals (*Phoca vitulina*), which are the most common seal species within the Skagerrak strait. There are at least seven harbour seal colonies in Skagerrak, one being located nearby Lysekil [48], while grey seals are far less frequent than harbour seals. Therefore, the 2 m– 4 m long seals were likely to be the grey seals (*Halichoerus grypus*) [49, 50]. Various species of cetaceans do occur on the Swedish west coast and among them are small-size cetaceans, the common bottlenose dolphin (*Tursiops truncatus*), the harbour porpoise (*Phocoena phocoena*), short-beaked common dolphin (*Delphinus delphis*), striped dolphin (*Stenella coeruleoalba*) and the white-beaked dolphin (*Lagenorhynchus albirostris*) [49, 50]. Large-size cetaceans that sometimes occurs on the Swedish west coast are orcas (*Orcinus orca*) and long-finned pilot whales (*Globicephala melas*) [49, 50]. The observed large marine mammals, e.g. in Fig 9, were likely an orca based on shape and size. The dorsal fin alone of the animal in Fig 9 measured 1.3 m, suggesting that this individual is a male orca, as the normal maximum size of dorsal fin in female orcas is ca. 0.9 m [51]. Apart from the information retrieved from the GBIF database, the authors were also verbally informed by the staff of the “The Sven Lovén Centre for Marine Sciences—Kristineberg” [52] and by other people in Lysekil, that an orca was observed swimming nearby the Gullmarn fjord during the time the data was collected. The four observations of orcas found in this study, were likely a reoccurrence of the same individual. These results emphasize the necessity of collecting data over longer periods and around the clock in order to follow up eventual risks and effects from marine renewables on marine animals, or general behaviour near marine renewables.

Observed fish were categorized as large fish [>40 cm– 90 cm], mid-size fish [20 cm– 40 cm] and small-size fish [<20 cm]. Large fish was likely Atlantic cod (*Gadus morhua*), a common species in Skagerrak [35, 36]. Juvenile and mature cod are known to use sandy bottom as feeding grounds specially in murky waters without vegetation [35, 36]. These conditions match those in the Lysekil research site in which photos (Fig 3C) and seabed samples were gathered and described in studies such as [31, 53, 54]. Mid-size fish were mostly observed in large schools and moving to avoid seals. This category of fish can either be the Northeast Atlantic mackerel (*Scomber scombrus*) or herring (*Clupea harengus*), both commonly occurring on the Swedish west coast [35, 36], and both species school in large numbers and are prey for e.g., seals. The smallest detected fish tentatively could be the European sprat (*Sprattus sprattus*). This species congregate in schools during daylight and tend to disperse at dark [43] as they are highly rely on vision to remain aggregated in schools [44], cohabitates shallow areas such as the surroundings of the Lysekil research site, and was frequently fished in this area before the commissioning of the research site. Seals also occurred simultaneously with schools of mid-size fish, suggesting their presence was associated with foraging. Seals swam either slow or rapid across the sonar FOV (Fig 11), and rapid swimming behaviour was more often observed in the presence of fish. We assumed that seals begun a more active hunting when their swimming behaviour changed from slow and steady to fast perusing mode. This result is similar to what [38] found in observations of periodic diving behaviour of harbour seals within the Skagerrak straight. Studies such as [38] described W-shaped dives as rapid vertical movements of seals to either pelagic or benthic depths where prey might be located. Although, the occurrence of seals was periodically distributed throughout the day, the hunting activity occurred mostly during the night from 19 until 6 UTC. Most of the observations of seals swimming in groups occurred in periods of daylight in which higher occurrence was found around 7, 12 and 16 UTC, respectively (Fig 12B). Similar patterns of seal foraging during night when food availability is high and resting during the day when foraging is less profitable were described at [55]. These results also strongly suggest that data collection over longer time periods are needed in order to better understand general behaviour near or around marine renewable energy installations.

Past studies showed that WECs may be an attractant to animals such as fish or crustaceans [56–61]. As fish attracting devices (FAD) creating a reefing effect they may also attract top predators such as marine mammals towards the device [62–64]. This matches our findings where schools of small and mid-size fish were repeatedly observed swimming near WEC A, and during daylight. On few occasions, it was possible to observe seals diving into large schools (Fig 14). The low frequency of observations of seals swimming close to the WECs can be related to the location of the sonar in relation to the WECs that may not have favoured the FOV for ease detection beyond the WECs. This can also be explained by the fact that the sonar platform landed in such way that the transducer’s pitch angle was favoring targets located at certain areas of the FOV. It can also be due to the pray (mid and large size fish) being mostly present within the wide space between the WECs. It even can be that the sonar platform due to its location and structure was attracting fish closer to it, more data is needed to explain this behaviour.

Earlier monitoring studies around marine renewables were conducted in the vicinity of hydrokinetic turbines, therefore limited information is available for comparison purposes between this (wave energy converters) and previous studies. For example [65] used a 200 kHz Split-beam sonar positioned sideways to monitor fish passing close to a horizontal axis helical turbine resulting in measurements of target strength and biomass. A combination of Split-beam (38/120/200 kHz) and imaging (260 kHz) sonar systems was used in another study [17] where shoals of fish and seabirds were observed occurring in the vicinity of horizontal axis tidal turbines. Schools of fish have also been observed in three-dimensions using a 400 kHz imaging sonar [66]. Similarly [67], used passive acoustic methods comprising arrays of hydrophones to monitor movements of small cetaceans in the vicinity of a tidal turbine, and was able to track harbour porpoise within 60 m from the turbine. The common aspects of these studies are the use of target strength and / or clicks to classify targets; moreover, these studies lack the measurements of target length, as well as the recognition of the shape of a target. One of the reasons leading to poor detection of shape and size of targets is the comparatively low operational frequency of these sonar systems. On the other hand, using sonar systems with higher frequency such as the MBS used in the present and other studies or underwater video cameras [46, 47, 67–73] can bring advantages in terms of shape and size of targets, but in the expense of a shorter effective range.

Marine renewable energy farms are restricted areas for human activities such as fishing and navigation and can sustain artificial reefs effects and increase bio productivity in the mid trophic hierarchy dominated by fish [6]. The present study was conducted when there was no noise and vibrations coming from the machinery. Therefore, the WECs could also be seen as a submerged pillar not too different to the submerged part of a monopile wind turbine, making this study comparable what can be expected within wind power farms. Marine energy farms do emit noise, vibrations and electromagnetic fields that can disturb marine animals [5, 47].

The present study also proves that multibeam imaging sonars can effectively be used for monitoring the occurrence and behaviour of species such as fish, seals, dolphins and other marine mammals around marine renewable energy devices but also in other marine monitoring. Moreover, the findings from this and other studies e.g. [47, 68, 70, 72, 73] can be considered as steps towards a standardised technique of monitoring marine energy sites using sonar systems.

In this study, we considered it more important to validate the monitoring technique based on imaging sonar systems rather than the actual metrics of the detected targets, therefore errors associated with the measurements may have been neglected. Neither can the present study be a reliable estimation of biomass. The study is a system calibration based on distributions of frequency of occurrence of targets in relation to time and in relation to the presence of marine renewable energy devices. Yet, estimated errors could be categorized as partially linked to the sonar’s own performance, and partially linked to the operator’s data handling and target counting procedures.

## 5. Conclusions

The use of acoustic images produced by multibeam sonar systems integrated into a standalone monitoring platform has proven to be an important around the clock tool for assessing the environment surrounding marine renewable energy devices over longer periods. The data collected by this platform also showed that different size categories of fish, seals and other marine mammals occur within the Lysekil research site could be recognised. Acoustic image technologies may therefore be an important tool for monitoring effects and eventual negative impacts on marine organisms, including those of economic importance, in relation to marine energy installations. However, species and biomass estimation studies that also include control areas need to be done in near future to cross compare the results. The occurrence of marine animals was distributed across characteristic time and space domains. Large fish [>0.4 m] frequently occurred at during night and near the benthic zone. Smaller fish frequently occurred during daylight and within the pelagic zone. The occurrence of seals was periodically distributed along a daily cycle, with intervals of 1–2 hours between maxima and minima. The research site comprises of several point absorber WECs, and based on the present observations, the conclusions are that fish, seals and large marine mammals can occur in marine renewable energy sites and may even be attracted. Although the WECs were not operating during the time of data collection, the results of this study give indication that fish and marine mammals can benefit from the existence of marine renewable energy farms.

As a future work, there is still much to be achieved with this technique of using imaging sonar systems for monitoring marine life within renewable energy farms. In this particular case, it is important to carry on observation of fish and marine mammals for longer periods at high temporal resolution and with operational WECs. It is also important to consider control sites so that biomass can be estimated in relation to the existence of WECs. Several additional sensors such as split-beam sonar, cameras and hydrophones can be integrated into a standalone, multifunctional platform enabling the acquisition of multidimensional data that can enhance the quantitative and qualitative analysis of the subsea environment surrounding marine renewable energy sites.

## Supporting information

S1 Data (XLSX)
